# Butein inhibits corticosterone-induced apoptosis of Neuro2A cells by maintaining MEK-ERK signaling

**DOI:** 10.1016/j.ibneur.2023.05.002

**Published:** 2023-05-08

**Authors:** Masanori Ohmoto, Masaya Takemoto, Tohru Daikoku

**Affiliations:** a Department of Pharmacy Practice and Sciences, Faculty of Pharmaceutical Sciences, Hokuriku University, Japan; b Department of Pharmaceutical Life Sciences, Faculty of Pharmaceutical Sciences, Hokuriku University, Japan

**Keywords:** Butein, Corticosterone, Apoptosis, Neuro2A cells, MEK, ERK, PI3K, AKT

## Abstract

Stress-induced overactivation of glucocorticoid signaling may contribute to mental illness by inducing neuronal death and dysfunction. We previously reported that pretreatment with the plant flavonoid butein inhibits corticosterone (CORT)-induced apoptosis of Neuro2A (N2A) cells. In the current study, we examined whether MEK-ERK and PI3K-AKT signaling pathways are involved in neuroprotection by butein. N2A cells were pre-incubated with serum-free DMEM containing 0.5 μM butein for 30 min, and then incubated with serum-free DMEM containing 0.5 µM butein, 50 µM CORT, 50 µM LY294002, or 50 µM PD98059 as indicated for 24 h. We subsequently performed the MTT assay and the western blot analysis. As expected, CORT considerably reduced N2A cell viability and increased relative expression of the apoptosis effector cleaved caspase-3, whereas pretreatment with butein blocked these cytotoxic effects. Treatment with CORT alone also decreased both AKT and ERK protein phosphorylation. Butein pretreatment had no effect on AKT phosphorylation, and only partially reversed the reduction in phosphorylated ERK. However, cotreatment with butein and the PI3K inhibitor LY294002 during CORT exposure enhanced ERK phosphorylation, whereas cotreatment with butein and the ERK phosphorylation/activation inhibitor PD98059 enhanced AKT phosphorylation, suggesting that MEK-ERK negatively regulates AKT phosphorylation. Moreover, the protective efficacy of butein was blocked by PD98059 cotreatment but not LY294002 cotreatment. These findings suggest that butein protects neurons against glucocorticoid-induced apoptosis by sustaining ERK phosphorylation and downstream signaling.

## Highlights

• Butein prevented Neuro2A cells from CORT-induced apoptosis by sustaining ERK phosphorylation.
• MEK-ERK signaling pathway negatively regulated AKT phosphorylation in N2A cells.

## 1 Introduction

**Fig. 1:**
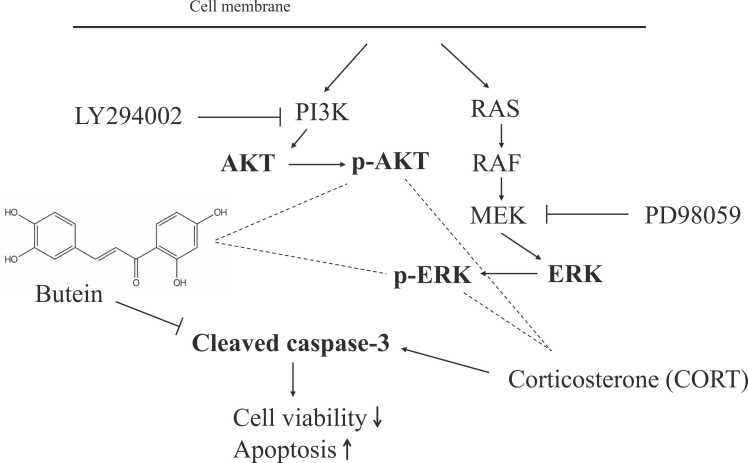
Schematic illustration of the mechanisms by which LY294002 and PD98059 inhibitors act on the PI3K-AKT and MEK-ERK pathways, respectively, in relation to butein's effects on CORT-induced cell viability and cleaved caspase-3 in Neuro2A cells.

Long-term elevations in glucocorticoid concentrations during chronic stress can induce hippocampal atrophy and impair hippocampal function, effects that are widely believed to contribute to the development of clinical depression. Depression constitutes a noteworthy concern in light of aging demographics and evolving social frameworks. The utilization of traditional botanical compounds to mitigate and prevent depression may present itself as a cost-efficient and low-risk alternative to pharmacological interventions. While possessing a notable bioactive profile of the plant flavonoid butein (Song et al., 2016, Padmavathi et al., 2017), few studies have focused on its antidepressant properties. Therefore, it is significant to scrutinize the potential efficacy of butein as a preventive and therapeutic measure against depression. In a previous study, we demonstrated that butein can prevent corticosterone (CORT)-induced Neuro2A (N2A) cell death as evidenced by enhanced viable cell number and reduced lactate dehydrogenase (LDH) release (Ohmoto et al., 2020). Concomitant with N2A cell death, CORT-induced intracellular reactive oxygen species (ROS) accumulation, reduced mitochondrial membrane potential, and increased expression of the apoptosis effector cleaved caspase-3. In addition, CORT increased nuclear expression of phosphorylated H2A histone family member X (H2Ax), a marker of apoptosis-related DNA damage. Recently, Tungalag et al. (2022) examined the impact of butein on oxidative stress in H9c2 cardiomyoblasts. The results indicated that butein increased cell viability and offered protection against oxidative damage by decreasing ROS generation, preventing mitochondrial dysfunction and apoptosis, and increasing antioxidant expression. All of these pro-apoptotic responses to CORT were suppressed by butein pretreatment. Butein modulates numerous intracellular signaling pathways related to oxidative stress and inflammation, but the mechanisms underlying suppression of CORT-induced apoptosis are unclear. The traditional medicinal herb component icariin has been reported to suppress CORT-induced neurotoxicity in primary culture at least in part by activation of the PI3K-AKT pathway (Zhang et al., 2012) and inhibition of p38MAPK phosphorylation (Liu et al., 2011). Liu et al. (2020) examined the effect of butein on SH-SY5Y cells exposed to microglia (BV2 cells)-conditioned medium, which serves as a model for Alzheimer's disease. They found that butein improves cell viability and reduces apoptosis in SH-SY5Y cells by regulating proteins related to ERK signaling pathways. This result suggests that butein may have a neuroprotective effect on microglial activation and neuroinflammatory injury. The RAS-MAPK and PI3K-AKT pathways are critical for cytoprotection under stress (Pettmann and Henderson, 1998, Marshall, 1995). To explore the plausible contributions of the MEK-ERK and PI3K-AKT pathways, we assessed the impact of butein on cell viability and cleaved caspase-3, which were induced by CORT in N2A cells. This evaluation was performed by obstructing ERK phosphorylation with the MEK inhibitor PD98059 and AKT phosphorylation with the PI3K inhibitor LY294002, as represented in Fig. 1.

## 2 Methods

### 2.1 Cell culture and drug treatment

Butein (Tokyo Chemical Industry, Japan), CORT (Fujifilm Wako, Japan), LY294002 (Fujifilm Wako), and PD98059 (Fujifilm Wako) were dissolved in dimethyl sulfoxide (DMSO) (Fujifilm Wako) and diluted in serum-free Dulbecco’s modified Eagle’s medium (DMEM, Fujifilm Wako) before application to N2A cell cultures. The final DMSO concentration was less than 0.1%. Control cultures were therefore incubated in 0.1% DMSO for all drug treatment experiments.

N2A cells were grown in 75 cm^2^ flasks containing DMEM supplemented with 10% fetal bovine serum and penicillin-streptomycin-amphotericin B solution (Fujifilm Wako) at 37 °C under a humidified 5% CO_2_ atmosphere. For drug treatment experiments, cells were reseeded on 96-well plates at 5000 cells/well and preincubated for 24 h. Cultures were washed with serum-free DMEM, incubated in serum-free DMEM containing butein (0.5 µM) for 30 min, and then incubated with serum-free DMEM containing CORT (50 µM), butein (0.5 µM), LY294002 (50 µM), or PD98059 (50 µM) as indicated for 24 h. The concentrations of LY294002 and PD98059 were based on a study by van der Heide et al. (2003). In some experiments, N2A cells were seeded at 5 × 10^5^ cells/flask in 25 cm^2^ flasks, grown to 80–90% confluence, treated as indicated, and lysed for western blotting. All other procedures and conditions were identical.

### 2.2 MTT cell viability assay

N2A cell viability was evaluated using 3-(4,5-dimethyl-2-thiazolyl)− 2,5-diphenyl-2 H-tetrazolium bromide (MTT) according to the manufacturer’s protocol (Dojindo Molecular Technologies, Japan). The absorbance of formazan generated from MTT by viable cells was measured at 535 nm using an Infinite 200 PRO microplate reader (Tecan, Switzerland). Cell viability was calculated relative to vehicle (DMSO)-treated control cultures (set at 100% viability) using the following equation: (A *treatment* – A *blank*) / (A *control* – A *blank*) × 100 (where, A = absorbance).

### 2.3 Western blotting

Cells cultured in 25 cm^2^ flasks were washed with phosphate buffered saline and cell lysates were prepared by adding RIPA lysis buffer (ATTO) containing a protease and phosphatase inhibitor. The cell lysate was passed through a 26 G needle several times to shear the DNA, thereby reducing lysate viscosity, and stored at − 80 °C. Total lysate protein was quantified using the Protein Assay BCA Kit (Fujifilm Wako). Briefly, lysate absorbance was measured at 562 nm on an Infinite 200 PRO microplate reader and values converted to protein concentrations according to a standard curve constructed using known ovine albumin concentrations. Lysates were then heat-treated with an equal volume of sample buffer, separated by sodium dodecyl sulfate (SDS)-polyacrylamide gel electrophoresis using 12.5% polyacrylamide gels (ATTO), and transferred to PVDF membranes (ATTO). After the transfer, blocking (buffer: TOYOBO, Japan) was performed overnight at 4 ºC. After blocking, the membranes were incubated with primary antibodies against phospho-ERK1/2, ERK1/2, phospho-AKT, AKT, and cleaved caspase-3 (1:1000, all from Cell Signaling Technology, USA) with a reaction solution (TOYOBO, Japan) at 4 °C overnight, washed with Tris-buffered saline plus 0.1% Tween 20 (TBST), probed with a horseradish peroxidase (HRP)-conjugated secondary antibody (Cell Signaling Technology) diluted 1:10,000 for 1 h at room temperature, and washed again in TBST. Target proteins were detected by chemiluminescence using Ez West Lumi One reagent (ATTO). Membranes were then stripped and probed with a peroxidase-conjugated anti-GAPDH antibody (1:1000, Fujifilm Wako). The expression levels of cleaved caspase-3 protein signals were normalized relative to the GAPDH signal to account for any differences in protein loading on the gel. Furthermore, we verified that the levels of AKT and ERK were correlated with GAPDH, which was used as the loading control.

### 2.4 Statistical analysis

All experimental values are presented as mean ± standard deviation of three independent experiments. Treatment group means were compared via analysis of variance followed by Tukey’s tests for between-group comparisons. A *P* < 0.05 (two-tailed) was considered statistically significant for all tests.

## 3 Results

### 3.1 Effect of CORT and butein on the phosphorylation of AKT and ERK in N2A cells

**Fig. 2:**
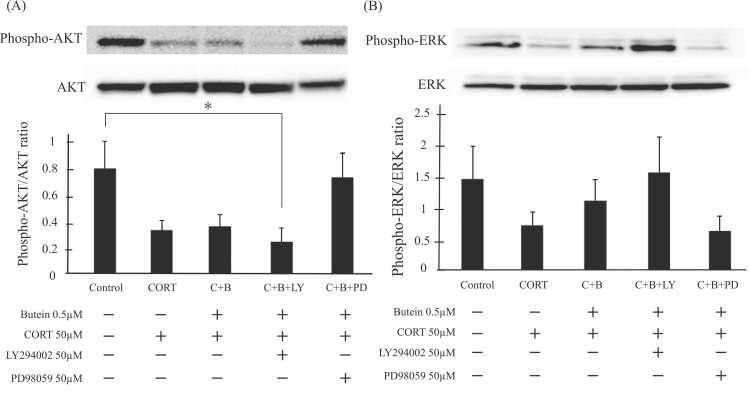
Butein maintains ERK signaling in Neuro2A cells during toxic CORT exposure. (A, B) Western blotting of (A) phosphorylated AKT and total AKT and (B) phosphorylated ERK and total ERK in lysates prepared from Neuro2A cells pretreated with 0.5 μM butein (C+B, C+B+LY, and C+B+PD) for 30 min and then cultured in DMEM containing the indicated drug combination for 24 h. The correlation between the expression levels of AKT and ERK and the level of expression of GAPDH as a loading control has been confirmed. C, B, LY, and PD represent CORT, butein, LY294002, and PD98059, respectively. Results are presented as the mean ± SD of three independent experiments (n = 3). * *P* < 0.05 vs. control.

Treatment of N2A cells with CORT markedly reduced the phosphorylated AKT (p-AKT) to total AKT expression ratio (p-AKT/AKT) compared to vehicle-treated control cultures, and this effect was not significantly altered by butein pretreatment and cotreatment (Fig. 2A). Pretreatment with butein and ensuing cotreatment with CORT, butein, and the PI3K inhibitor LY294002 slightly reduced p-AKT/AKT compared to cultures pretreated with butein and cotreated with CORT plus butein, and significantly reduced p-AKT/AKT compared to vehicle-treated control cultures. In contrast, pretreatment with butein and cotreatment with butein, CORT, and the ERK inhibitor PD98059 markedly enhanced the p-AKT/AKT compared to cultures pretreated with butein and cotreated with CORT and butein, suggesting that p-ERK negatively regulates AKT phosphorylation. Thus, we speculated that butein increases ERK phosphorylation and activation but has little direct effect on AKT phosphorylation and activation under CORT toxicity.

We confirmed these putative effects of butein by directly measuring the p-ERK/ERK ratio (Fig. 2B). Treatment with CORT alone reduced p-ERK compared to vehicle-treated controls, whereas pretreatment and cotreatment with butein enhanced p-ERK/ERK compared to cultures treated with CORT alone. Further, pretreatment with butein and cotreatment with CORT, butein, and LY294002 increased p-ERK compared to cultures pretreated with butein and cotreated with butein and CORT, whereas PD98059 cotreatment slightly decreased p-ERK. Thus, butein appears to prevent the CORT-induced decrease in p-ERK. Furthermore, ERK activity may negatively regulate AKT phosphorylation and signaling activity. These findings, particularly the increase in p-AKT following PD98059 cotreatment, suggest reciprocal negative regulation of MEK-MAPK and PI3K-AKT signaling pathways.

### 3.2 Effects of CORT and butein on N2A cell viability and mediation by MEK-ERK and PI3K-AKT signaling pathways

**Fig. 3:**
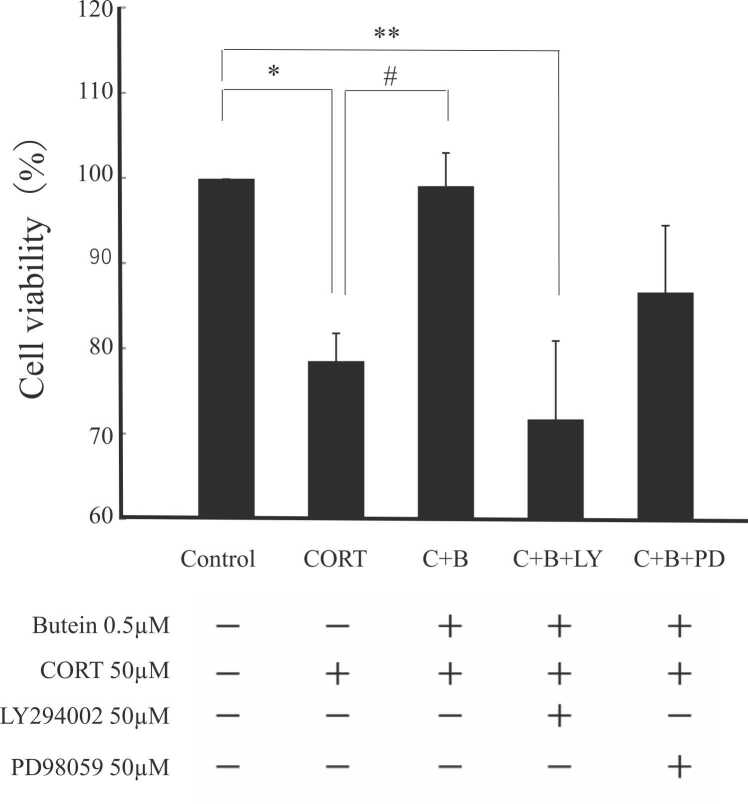
Butein protects Neuro2A cells against CORT-induced toxicity. Cells were treated with 0.5 μM butein (C+B, C+B+LY, and C+B+PD) for 30 min followed by treatment with the indicated drug combination for 24 h. Total viable cell number was then estimated by MTT assay. C, B, LY, and PD represent CORT, butein, LY294002, and PD98059, respectively. Results are presented as the mean ± SD of three independent experiments (n = 3) with three technical replicates. * *P* < 0.05 vs. control, ** *P* < 0.01 vs. control, # *P* < 0.05 vs. CORT treated group.

As demonstrated previously, CORT treatment reduced N2A cell viability and this effect was reversed by butein pretreatment (Fig. 3). In our previous study, we confirmed that butein at 0.5 μM showed no toxicity and no significant difference from the control group. In contrast, cotreatment with LY294002 significantly reduced the protective efficacy of butein as evidenced by cell viability near that of the CORT alone group. However, cotreatment with PD98059 did not markedly reduce the protective efficacy of butein as cell viability remained significantly higher than that of the CORT alone group. We found no difference in cell viability between the group co-treated with LY294002 and PD98059, and the group treated with CORT alone. Therefore, we concluded that LY294002 and PD98059 did not affect cell survival or toxicity more than the effects observed with CORT. This result implies that butein does not inhibit the CORT-induced decrease in phosphorylation of AKT (Fig. 2 A). However, it maintained the p-ERK/ERK ratio of samples cotreated with butein and CORT compared to those treated with CORT alone (Fig. 2B). Consequently, these findings suggest that butein protects N2A cells against CORT toxicity by maintaining phosphorylation of ERK and activation of downstream signaling pathways.

### 3.3 Effects of butein and CORT on cleaved caspase-3 and mediation by MEK-ERK and PI3K-AKT signaling pathways

**Fig. 4:**
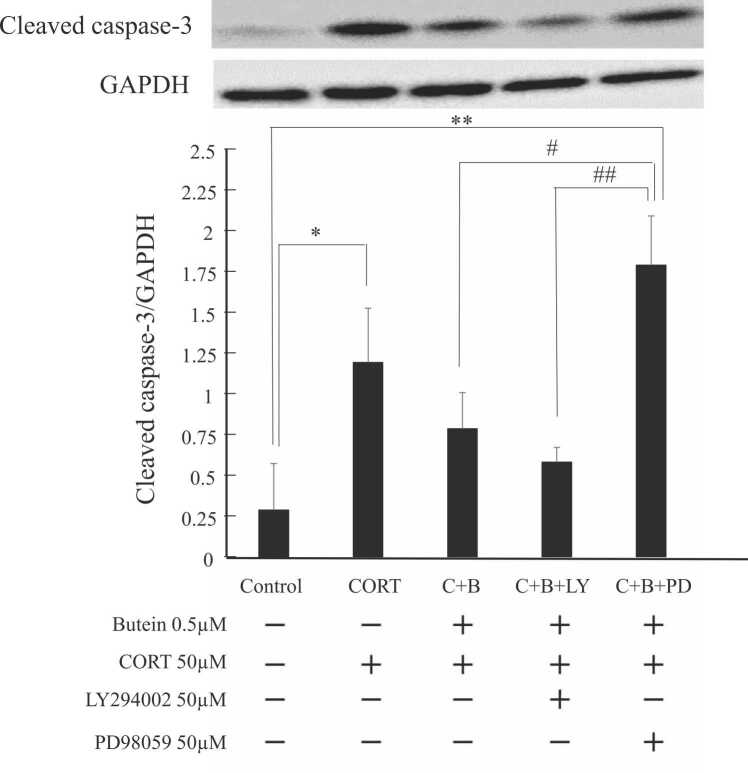
Butein reduces CORT-induced apoptosis by enhancing ERK signaling. Neuro2A cells were pretreated with 0.5 μM butein (C+B, C+B+LY, and C+B+PD) for 30 min and then with the indicated drug combination for 24 h, followed by western blotting for cleaved caspase-3. C, B, LY, and PD represent CORT, butein, LY294002, and PD98059, respectively. Results are presented as the mean ± SD of 3 independent experiments (n = 3). GAPDH expression was measured as the gel loading control. * *P* < 0.05 vs. control, ** *P* < 0.01 vs. control, # *P* < 0.05 vs. PD98059 cotreated group, ## *P* < 0.01 vs. PD98059 cotreated group.

Treatment with CORT alone significantly increased the cleaved caspase-3/GAPDH ratio in N2A cells compared to the control group, consistent with induction of apoptosis (Fig. 4). Butein pretreatment reduced the cleaved caspase-3/GAPDH ratio compared to the CORT alone treatment group, although the difference did not reach statistical significance. Cotreatment with LY294002 and butein during CORT exposure slightly increased the cleaved caspase-3/GAPDH ratio compared to vehicle treatment (control) and butein pretreatment, whereas PD98059 significantly enhanced the cleaved caspase-3/GAPDH ratio compared to the butein pretreatment group. These findings suggest that AKT phosphorylation has little effect on cleaved caspase-3, whereas ERK inhibition markedly increases cleaved caspase-3. Therefore, the protective effect of butein on CORT-induced apoptosis appears to be mediated by activation of MEK-ERK signaling.

## 4 Discussion

We demonstrate that the flavonoid butein found in a variety of flowering plants protects against corticosterone-induced neuronal apoptosis by sustaining ERK phosphorylation. Treatment of N2A cells with CORT for 24 h induced apoptosis as evidenced by a reduction in viable cell number and increased intracellular expression of cleaved caspase-3, whereas butein (pretreatment and cotreatment) reversed these signs of apoptosis. The protective effect of butein was blocked by inhibition of ERK phosphorylation, suggesting that butein protects neurons from CORT-induced apoptosis by promoting activation of the MEK-ERK signaling pathway. Furthermore, we found evidence for reciprocal negative regulation between MEK-MAPK and PI3K-AKT/PKB signaling pathways in N2A cells, as AKT phosphorylation was increased by the ERK phosphorylation inhibitor PD98059 and inhibition of AKT activity by LY294002 slightly enhanced p-ERK expression. However, while inhibition of ERK phosphorylation also reduced cell viability and increased cleaved caspase-3 expression during CORT treatment, blockade of AKT phosphorylation did not alter the cytotoxic effects of CORT. Thus, negative regulation of AKT phosphorylation by ERK may not contribute to the neuroprotective efficacy of butein.

In accordance with our findings, van der Heide et al. (2003) reported crosstalk between PI3K-AKT/PKB and Ras-Raf-MEK-ERK1/2 pathways in N2A cells following PI3K-AKT activation by insulin receptor stimulation. Insulin promoted AKT phosphorylation in a dose-dependent manner, and concomitant treatment with the PI3K inhibitor LY294002 suppressed AKT/PKB phosphorylation, indicating that insulin activates PI3K-AKT/PKB. Conversely, insulin dose-dependently inhibited ERK1/2 and this effect was not altered by the MEK inhibitor PD98059, but the insulin-induced phosphorylation of ERK1/2 was restored by PI3K inhibition using LY294002. These findings suggest strong mutual regulation of PI3K-AKT/PKB and Ras-Raf-MEK-ERK1/2 pathways downstream of the insulin receptor, possibly through AKT/PKB-mediated inhibition of the ERK signaling component Raf. Thus, butein suppresses cellular apoptosis by upregulating ERK phosphorylation, but suppresses p-AKT expression through signal crosstalk. It is currently unclear if this latter effect contributes to other documented bioactivities of butein.

Zhang et al. (2012) also reported that CORT reduced the viability of primary cultured rat hypothalamic neurons and concomitantly increased cleaved caspase-3 expression, underscoring the broad cytotoxicity of glucocorticoid overstimulation. Further, these authors reported that another plant flavonoid, icariin, reduced CORT cytotoxicity, but that this effect was mediated by activation of the PI3K-AKT pathway. Exposure of neurons to CORT triggered intracellular ROS accumulation and caspase-3 activation, and reduced mitochondrial function, ultimately leading to cell death as evidenced by LDH release, whereas pretreatment with icariin increased phosphorylated AKT levels and significantly inhibited CORT-induced oxidative stress and caspase-3-dependent apoptotic signaling. Liu et al. (2011) also reported that CORT treatment dose-dependently induced apoptosis in primary cultured rat hippocampal neurons as evidenced by terminal deoxynucleotidyl transferase dUTP nick end labeling (TUNEL), whereas treatment with icariin significantly reduced the number of TUNEL-positive cells. However, the protective effect of icariin appeared to involve inhibition of p38MAPK phosphorylation rather than phosphorylation of ERK and JNK1. Therefore, these plant flavonoids may protect against CORT-induced neurotoxicity through distinct mechanisms. The protective effect of butein in a neuroinflammation model has been examined (Liu et al., 2020). SH-SY5Y neuroblastoma cells were treated with conditioned medium from BV2 microglia cells exposed to lipopolysaccharide and pretreated with butein. The results indicated that butein pretreatment increased cell viability and reduced apoptosis in the cells. It also reversed mRNA expression and protein phosphorylation related to upregulated ERK signaling pathways and reduced NF-κB p65 transcriptional activity in the cells. These findings suggest that butein effectively mitigates the negative consequences of microglial activation and plays a neuroprotective role by blocking the ERK signaling pathway and reducing the activity of NF-κB. In our previous study (Ohmoto et al., 2020), CORT treatment increased LDH release as well as cleaved caspase-3 expression, consistent with the present study. Loss of mitochondrial membrane potential, elevated intracellular ROS levels, and accumulation of γH2AX, a marker of DNA damage, were also detected. Loss of mitochondrial membrane potential, efflux of cytochrome c into the cytoplasm, formation of a complex, including APAF-1 and caspase-9, and the ensuing cleavage of caspase-3 is a major final common pathway leading to apoptosis (Pettmann and Henderson, 1998). Corticosterone may induce this cell death process by triggering oxidative stress and mitochondrial dysfunction, while butein may prevent apoptosis by enhancing antioxidant capacity via ERK activation. Indeed, ERK activation is required for the nuclear translocation of nuclear factor erythroid 2–related factor 2 (Nrf2), a master regulator of the cellular antioxidant response (Chen et al., 2017, Lin et al., 2010). It is also possible that downstream changes in the balance between pro-apoptotic BAX and anti-apoptotic BCL-2 are involved in this cytoprotection by butein.

The main limitation of the current investigation was the exclusion of primary cultures of murine neurons to avoid the use of animals, and instead utilizing N2A cells (i.e. neuroblastoma). This approach provides ease of cultivation and enables consistent and reproducible outcomes across different laboratories. N2A cells are a well-established neuronal model that can differentiate into a neuronal-like phenotype and form neurites through serum reduction and retinoic acid treatment (Bulfone et al. 2005). These cells are commonly used in studies related to neuronal differentiation and neurotoxicity. Our previous study (Ohmoto et al. 2020) showed that butein inhibited the suppression of neurite outgrowth in N2A cells caused by CORT. The differentiated N2A cells are only a substitute for normal neuronal cells, and therefore, future studies should validate our initial findings using primary neuronal cells. Another apprehension is that our investigation solely relied on changes in protein levels of phosphorylated proteins and cleaved caspase-3, which were evaluated through western blotting exclusively. To ensure the accuracy of our findings, it is crucial to validate them using alternative methods, such as enzyme-linked immunosorbent assays and immunocytochemistry. Particularly, immunocytochemistry provides an advantageous means of confirming western blotting results by allowing for the visualization of protein expression within the cell.

## 5 Conclusions

The neuroprotective properties of butein against corticosterone-induced apoptosis suggest its involvement in the maintenance of ERK phosphorylation. Additionally, it indirectly attenuates AKT phosphorylation through reciprocal negative regulation between the PI3K-AKT and MEK-ERK pathways. However, it remains uncertain if the latter effect contributes to the bioactivity of butein. Hence, butein may serve as an effective adjunct therapy for stress-induced neuronal damage.

## CRediT authorship contribution statement

M.O. designed the study, carried out the experiments, performed statistical analyses, and drafted the manuscript. M.T. and T.D. participated in the design of the study. All authors red and approved the final manuscript.

## Declaration of Competing Interest

All authors declare having no competing interests.
